# Direct Observation of Propagating Spin Waves in a Spin Hall Nano‐Oscillator

**DOI:** 10.1002/adma.74547

**Published:** 2026-08-28

**Authors:** Victor H. González, Frank Schulz, Nilamani Behera, Martina Ahlberg, Akash Kumar, Andreas Frisk, Felix Groß, Sven Erik Ilse, Steffen Wittrock, Markus Weigand, Gisela Schütz, Johan Åkerman, Sebastian Wintz

**Affiliations:** ^1^ Department of Applied Mathematics and Theoretical Physics University of Cambridge Cambridge UK; ^2^ Department of Physics University of Gothenburg Gothenburg Sweden; ^3^ Max‐Planck‐Institut für Festkörperforschung Stuttgart Germany; ^4^ Max‐Planck‐Institut für Intelligente Systeme Stuttgart Germany; ^5^ Department of Physics, School of Basic Sciences Indian Institute of Technology Bhubaneswar Odisha India; ^6^ Helmholtz‐Zentrum Berlin für Materialien und Energie GmbH Berlin Germany; ^7^ Research Institute of Electrical Communication (RIEC) Tohoku University Sendai Japan; ^8^ Center for Science and Innovation in Spintronics (CSIS) Tohoku University Sendai Japan

**Keywords:** spin Hall nano‐oscillators, spin waves, spintronics, STXM

## Abstract

Constriction‐based spin Hall nano‐oscillators (SHNOs) show great promise for application as highly tunable microwave sources with straightforward scalability toward large coupled networks. However, details of the magnetization dynamics within SHNOs have thus far not been addressed experimentally, due to the minute time and length scales involved. In this work, we present direct imaging of the magnetization dynamics within a single CoFeB‐based SHNO using time‐resolved scanning transmission x‐ray microscopy (TR‐STXM). Our measurements reveal that the magnon amplitude is strongest at the two constriction edges, with a pronounced asymmetry favoring one edge, and that the emitted spin waves (SWs) exhibit strongly anisotropic propagation. Micromagnetic simulations suggest that grain boundaries and the Dzyaloshinskii–Moriya interaction (DMI) play a key role in both effects. Furthermore, the magnetodynamics changed during measurement, indicating that the CoFeB/MgO interface may be more susceptible to x‐ray‐induced modifications than previously recognized, challenging its presumed radiation hardness.

## Graphical abstract

By combining frequency injection locking with time‐resolved scanning transmission x‐ray microscopy (TR‐STXM), the authors image the real‐time magnetization dynamics of a spin Hall nano‐oscillator (SHNO) for the first time. Their results reveal richer dynamics than previously reported and challenge existing assumptions regarding the radiation hardness of the CoFeB/MgO interface.

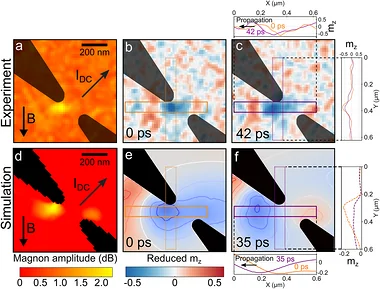

## 1 Introduction

The generation and manipulation of collective spin excitations is at the heart of spintronics and magnonics, owing to their potential to enable low‐consumption processing of digital and analog information [1, 2], and next‐generation low‐power non‐Von Neumann computational architectures [3, 4]. These collective spin excitations, or spin waves (SWs), are able to propagate (and carry information) without Joule heating. Building on this low‐consumption propagation, SWs have been explored for miniaturized delay lines [5], Boolean logic [6, 7, 8], signal processing [9], and wave‐based unconventional computing [10, 11, 12, 13, 14].

Nanoconstriction‐based spin Hall nano‐oscillators (SHNOs) are one of the most promising and versatile devices for SW generation given their simple and mature fabrication process [15], CMOS compatibility [16], and voltage tunability [15, 17, 18, 19]. They support multiple dynamical modes, both localized [20] and propagating [21, 22], and convert a direct current (DC) input into a tunable radio‐frequency (RF) output, making them ideal for exploring diverse SW phenomena within a single device. This versatility has also enabled the construction of SHNO arrays susceptible to mutual synchronization, which not only provides a model platform for studying complex dynamics in coupled nonlinear systems [23], but also improves linewidth and output power, facilitating tunable RF applications [24]. Furthermore, SHNO arrays might also serve as a potential platform for low‐power, highly scalable neuromorphic SW computing [18, 24, 25, 26].

A detailed understanding of the magnetization dynamics within SHNOs requires experimental techniques capable of resolving nanometer spatial scales and sub‐nanosecond temporal dynamics. Conventional approaches (such as Brillouin light scattering (BLS), electrical detection via anisotropic magnetoresistance (AMR), or micromagnetic simulations) have each provided valuable insights into SHNO behavior. However, BLS is limited in spatial resolution, AMR offers only indirect access to the dynamics, and simulations, while powerful, cannot fully replace experimental validation.

In contrast, time‐resolved scanning transmission x‐ray microscopy (TR‐STXM) enables direct visualization of nanoscale magnetization dynamics, capturing both spatial profiles and temporal evolution of SW modes. Even static STXM has revealed SW features not anticipated by theory: the unexpectedly large size of magnetic droplet solitons and their controlled freezing and thawing being such an example [27, 28]. Therefore, extending STXM to the time domain provides an essential tool for understanding the complex behavior of individual oscillators, which, in turn, governs their coupling in arrays. For instance, recent work on SW‐mediated SHNO synchronization has shown that the wavelength and propagation direction of SWs critically influence the phase relationships between neighboring oscillators [29]. Direct observation of these dynamics therefore provides the missing link needed to accurately model, design, and optimize coupled SHNO systems.

In this work, we directly observe SW auto‐oscillation (AO) modes within a constriction‐based SHNO using time‐resolved STXM. This observation allows us to reevaluate many assumptions of previous studies and shed new light on the emission mechanisms of SWs in SHNOs.

## 2 Results and Discussion

### 2.1 Direct Observation of Auto‐Oscillation Modes

**FIGURE 1:**
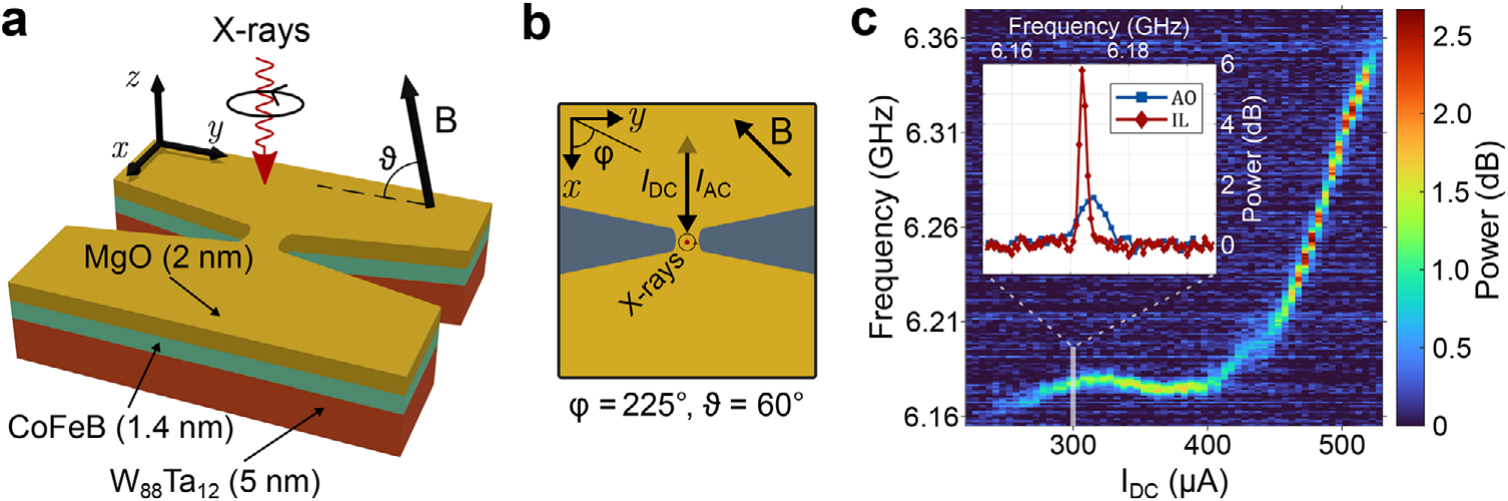
Sample and electrical measurement. (a) 3D schematic of the SHNO device showing the orientations of the external field and incident x‐rays. The external field **B** had a strength of 255mT, in‐plane angle 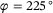, and out‐of‐plane angle 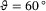. The x‐ray incident angle was normal to the sample plane. (b) Sample top view. (c) Power spectral density (PSD) of the AOs as a function of $I_{\mathrm{DC}}$. The inset shows a cut along the white line of the free‐running (blue squares) and injection‐locked (red diamonds) signal at 300μA.

The samples, constriction‐based SHNOs, were fabricated from $W_{88}$ ${\mathrm{Ta}}_{12}$(5 nm)/${\mathrm{Co}}_{20}$ ${\mathrm{Fe}}_{60}$ $B_{20}$(1.4 nm)/MgO(2 nm) trilayer stacks, and the device design is illustrated in Figure 1a,b. A direct current ($I_{\mathrm{DC}}$) running through the W‐Ta heavy metal (HM) generates a spin‐orbit torque (SOT) in the CoFeB layer via the spin Hall effect. In the constriction region, the SOT is large enough to compensate damping and thereby generate a localized magnetization precession called AO [30]. The MgO layer is used to induce perpendicular magnetic anisotropy (PMA), which is necessary to excite propagating SWs [17, 21]. A detailed description of the fabrication process is provided in the Section 4.

Figure 1c shows the power spectral density (PSD) of the SHNO AO as a function of $I_{\mathrm{DC}}$ in an external field of 255mT, applied at oblique in‐plane (IP) (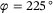) and out‐of‐plane (OOP) (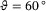) angles. The onset of AO is observed at approximately 220μA, showing a non‐monotonic current dependence of the AO frequency, in the range between 220 and 460μA.

This non‐monotonic behavior is a characteristic feature of SHNOs with PMA and has been associated with AOs localized close to the constriction due to a negative nonlinearity coefficient, which describes the magnitude and sign of magnon–magnon interactions in the system [21, 31]. At higher currents, the AO frequency ($f_{\mathrm{AO}}$) undergoes a blue shift. This is evidence of a modified confinement of the SW mode. As more SOT is exerted (larger $I_{\mathrm{DC}}$), the magnetization precesses at a larger angle, reducing the demagnetizing field and changing the sign of the magnon–magnon nonlinearity. This change in sign promotes propagation of the SWs outside the nanoconstriction region and a quasi‐linear increase in AO frequency [21].

The SHNO dynamics can be further stabilized using a process known as second harmonic injection locking. In this process, an external signal with twice the frequency of the AO mode is injected. This signal phase‐locks and drives the oscillator at $f_{\mathrm{AO}}$, greatly increasing its power and reducing its linewidth [32]. This is illustrated in Figure 1c, which shows the signal with and without IL at $I_{\mathrm{DC}}=300\;\mu A$. The signal was background corrected by subtracting a spectrum recorded at zero magnetic field and zero current. By injection‐locking the device, we are not only drastically increasing the signal strength that can be detected, but also enabling TR‐STXM. The operation frequency of the microscope can be synchronized with the AO frequency, allowing time‐resolved detection of the magnetization dynamics. The details of the electrical and TR‐STXM setups are given in  Section 4.

The TR‐STXM measurement was performed at a nominal temperature of 50K and a 300μA driving current. The applied field was kept at the same strength and orientation (**B** =255mT, 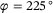, and 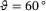). The AO, and hence the imaged dynamics, was at $f_{AO}=6.17\;\mathrm{GHz}$, in accordance with the electrical characterization in Figure 1c. Injection locking was applied at twice this frequency, that is, at $f_{IL}=2f_{AO}=12.34\;\mathrm{GHz}$.

**FIGURE 2:**
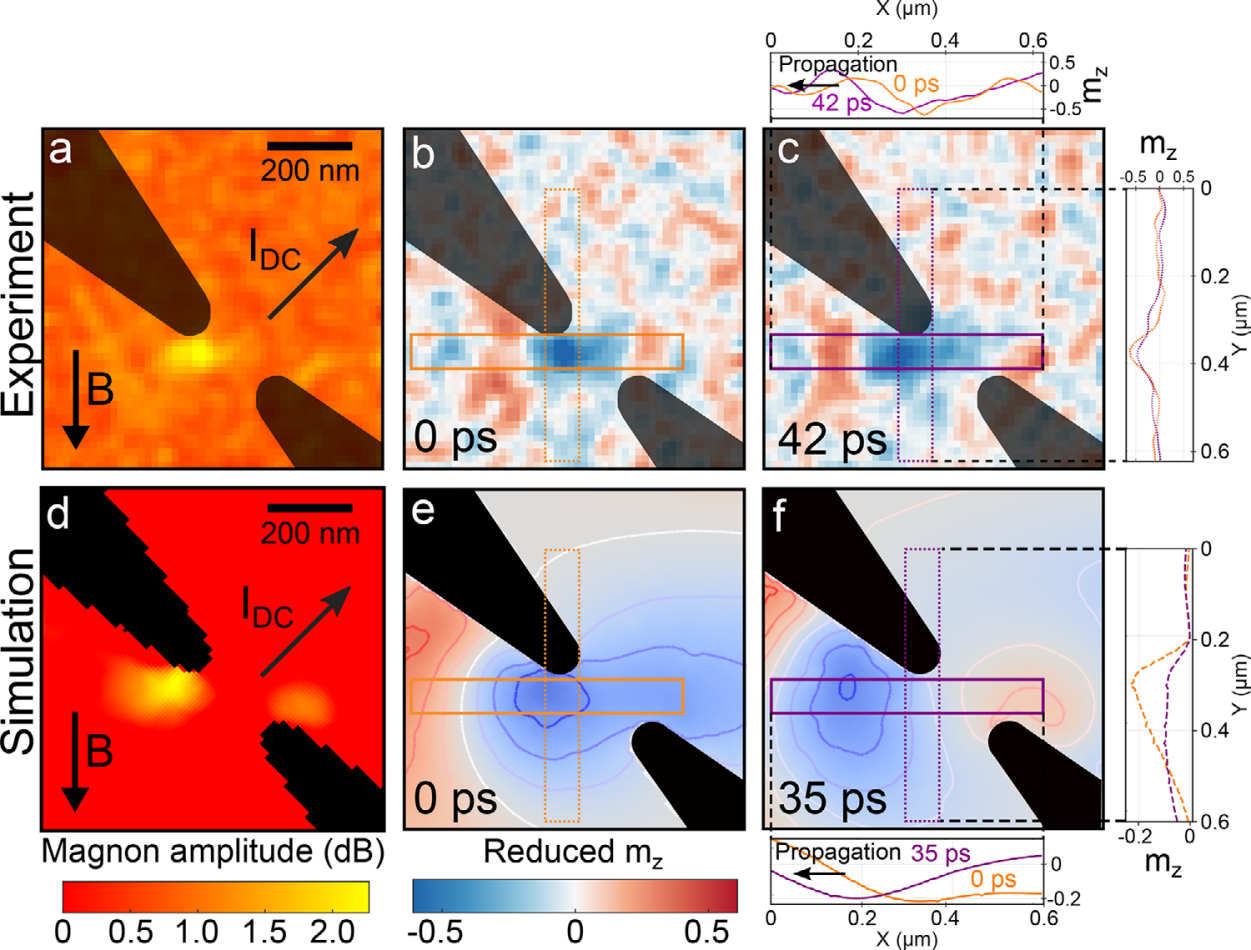
Experimental and simulated ps‐fast magnetization dynamics. (a) Measured relative magnon amplitude. The highly asymmetrical amplitude suggests the presence of additional magnetic interactions, not considered by the existing literature. (b,c) Snapshots of the spin wave propagation. Average magnetization ($m_{z}$) of the marked rectangular areas is shown in arbitrary units in the insets to the right and top. We observe a spin wave propagating to the left in the horizontal direction. (d–f) Simulated magnon amplitude and snapshots. Agreement between the experiment and the simulation was only achieved with the inclusion of grain boundaries and DMI in the CoFeB layer.

The dynamic OOP magnetization $m_{z}$ was measured over an $800\times 800\;\;nm^{2}$ square‐shaped area around the nanoconstriction. The acquired 31 frames were processed using an FFT‐based algorithm to obtain frequency‐specific space‐resolved magnon amplitude and phase of the signal, which can be looked at separately or recombined into snapshots. Figure 2a shows the amplitude map at the AO frequency. We observe the presence of a SW mode localized at the edges of the constriction. These edge modes are characteristic of SHNOs with [21] and without [31] PMA, but our observation shows mode asymmetry that has not been previously reported.

The phase information is included in the snapshots in Figure 2b,c showing two different points in time. The insets at the top and the side of Figure 2c display the average magnetization in the areas of the horizontal and vertical rectangles, respectively. A directed SW propagation perpendicular to the applied field can be observed, with the wavefront moving horizontally to the left (upper inset). Parallel to **B**, the wave remains in the same position (side inset). Animations of the time‐resolved data and the corresponding simulation (discussed below) are provided in the Supporting Information, as detailed in Section S.2.

The anisotropic propagation is surprising and highlights previously undetected features of the local magnon potential landscape in SHNOs. The observation has significant implications, as it challenges existing assumptions and may prompt a reevaluation of our fundamental understanding of the intrinsic dynamics of a single oscillator, ultimately affecting how we model, design, and optimize SHNO‐based devices.

### 2.2 Anisotropic Spin Wave Propagation

The direct imaging of the SW modes reveals unexpected localization and propagation asymmetries. To better understand these features, we modeled the measured SW dynamics using micromagnetic simulations, as demonstrated in Figure 2d–f. All parameters of the model are provided in Section 4. In order to reproduce the observed magnetization dynamics, we had to lower the measured (blanket film) PMA, add grain boundaries, and include interfacial Dzyaloshinskii–Moriya interaction (DMI) in the magnetodynamical layer. With these ingredients, the simulations reproduce the measured mode shape, propagation direction, and asymmetry well (Figure 2); only the precise timing of the experimental and simulated snapshots differs slightly. This level of discrepancy is expected, since the exact parameters of an individual SHNO cannot be directly measured, but must be adjusted in simulations. To assess the effect of each contribution on the shape of the SW modes, we performed a systematic study in which these components were added to the model sequentially.

**FIGURE 3:**
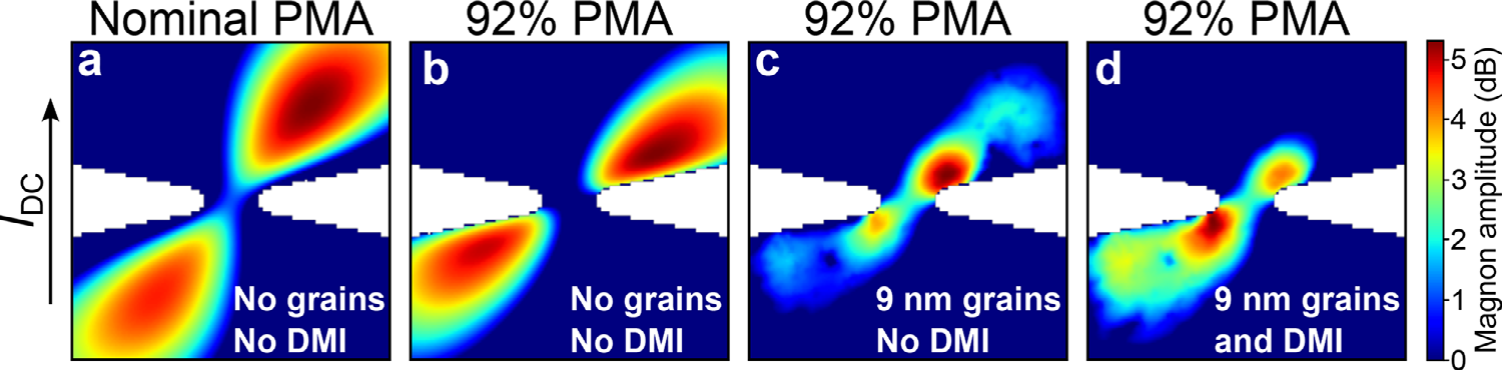
AO modes for different simulation parameters. (a) Nominal PMA and monocrystalline film, similar to the existing literature. (b) Reduced PMA. (c) Reduced PMA with grain boundaries. (d) Reduced PMA with grain boundaries and DMI. In order to reproduce the emission asymmetry and the preferred SW emission direction observed in the measurements, grain boundaries and DMI must be considered. $I_{\mathrm{DC}}$ flows in the direction indicated by the arrow on the left.

Figure 3a shows the simulated magnon amplitudes for a CoFeB layer based on the existing literature [17, 21, 33] and the experimental PMA value (blanket film, before fabrication). Although there is a slight amplitude asymmetry, the shape of the modes is symmetrical and extended, as expected for propagating SW edge modes. In contrast, the modes observed in the synchrotron measurement are more localized, just off the side of the constriction, and have a more pronounced IP propagation anisotropy (Figure 2).

By refining the PMA value in our simulations (Figure 3b–d), the modes start to resemble the measurement. Weaker PMA in devices compared to the as‐grown film has been observed before in SHNOs, and the effect is more pronounced for smaller nanoconstrictions [21]. We suspect that the fabrication procedure, specifically the Ar ion milling, affects the CoFeB/MgO interface and thereby reduces the PMA. It has been reported that ion irradiation can introduce defects at CoFeB/MgO interfaces and reduce the PMA [34, 35, 36], which is known to be sensitive to random rearrangements of the crystalline lattice [37]. Other possible mechanisms are Ta diffusion into the CoFeB [38] and oxidation of the magnetodynamical layer at the patterned edges [39]. Regardless of the PMA reduction's origin, the SW modes are very susceptible to the local magnetic environment, and modifications of the anisotropy landscape certainly modify the localization and propagation direction of the AO modes, as seen in Figure 2a,b. In the simulations (Figure 3b), an 8% decrease in PMA delocalizes the oscillating modes from the constriction region, which displaces the origin and propagation direction of the SWs. The specific percentage number was found by performing micromagnetic simulations with different PMA values and matching the AO mode profiles with the experiment.

Previous simulations on realistic polycrystalline constriction‐based SHNOs found that the presence of grain boundaries indeed changes the AO modes [40]. In this work, the presence of grain boundaries lowers the current threshold for the onset of AOs. A simulated monocrystalline device requires 350μA to auto‐oscillate, in contrast to the less than 300μA necessary both experimentally and in our final simulations. More importantly, the shortened propagation length due to magnon scattering at the grain boundaries localizes the edge modes and sharply increases the difference in relative power between the two edges. In the measurement, the low‐amplitude mode can easily be missed due to the low signal‐to‐noise ratio. However, it is present upon careful observation of Figure 2b,c. The dominant SWs (appearing mainly as blue at the depicted instant) propagate to the left, while weaker waves with inverted phase (red) propagate in the opposite direction, as seen at the right end of the horizontal rectangles.

Figure 2a also displays a faint structure at the predicted position. Our average grain diameter of 9nm (found by simulating different grain sizes) (Figure 3c) is consistent with the previously reported experimental value for similar ${\mathrm{Co}}_{60}$ ${\mathrm{Fe}}_{20}$ $B_{20}$ thin films [41].

It is well established that HM‐ferromagnet heterostructures lead to DMI (D) [42, 43, 44, 45]. Yet, DMI is most often neglected in experimental and numerical treatments of nanoconstriction SHNOs. In our simulations, we used an experimental value of D= $250\;\mu J/m^{2}\;$ reported for ultrathin W/CoFeB/MgO heterostructures [45], and the results show an excellent match to the measurement (Figure 2).

When DMI is included in the model (Figure 3d), the asymmetry of the SW emission is inverted with respect to the current direction, and the asymmetry of the edge modes becomes more pronounced. To be discerned, these features require the detailed information obtained from direct imaging. This explains why, although DMI has been mentioned in previous SHNO studies [17, 46], this is the first time it must be explicitly included in order to reproduce the experimental results. Consequently, TR‐STXM has allowed us to develop a more nuanced understanding of the operation of real‐world devices in a way that previous indirect or time‐integrated measurements could not. In turn, we expect that these results can guide SHNO design and fabrication for microwave and neuromorphic applications.

### 2.3 Irreversible X‐Ray Effects on CoFeB Magnetodynamics

Exposure to x‐rays irreversibly alters the CoFeB layer, making a systematic study of the magnetodynamics challenging. Several samples with different compositions and thicknesses of the CoFeB layer have been studied, and in each case it was found that the AO curve changes significantly upon x‐ray illumination. Both the shape as well as the general frequency range changed, which was often accompanied by a decrease in AO amplitude. The signal strength briefly increased before decreasing steadily, and suddenly dropping.

**FIGURE 4:**
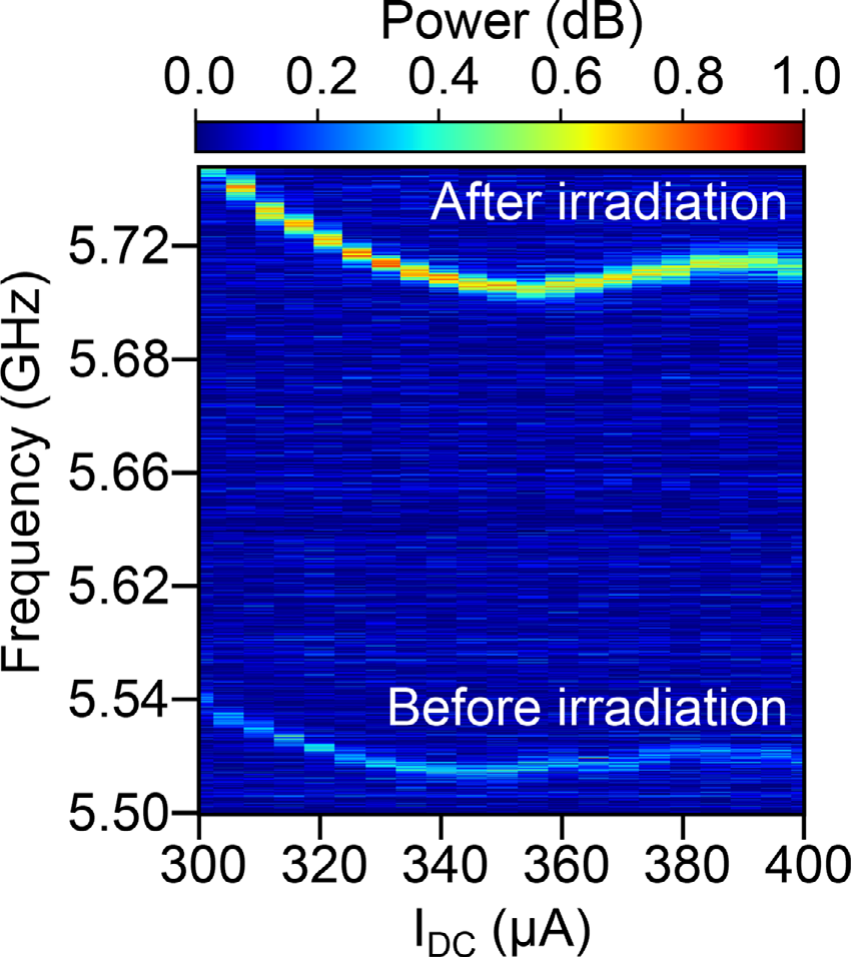
AO modes before and after x‐ray irradiation. Power spectral density as a function of current. The frequency and amplitude increase after irradiation, hinting at larger values of the PMA and SOT. The modified curvature implies a transformation of the mode geometry. While a rising frequency was consistently observed, the changes in amplitude and curvature varied between samples.

Figure 4 depicts an example of x‐ray induced changes in the magnetodynamics. Note that this was not the same SHNO as in Figures 1c and 2. The original device did not exhibit any dynamics/electrical signal after substantial x‐ray exposure. The AO frequency rises by hundreds of MHz, and both the amplitude and curvature increase. However, the increase of the latter two is not consistent across all samples; instead, they often decrease when irradiated, owing to device‐to‐device variability. Similar soft x‐ray‐induced modifications—yet possibly reversible—have been reported for other magnetic systems [47]. The acquisition of TR images (Figure 2) was performed before major degradation occurred, ensuring the reliability of the observed AO modes. Long‐term imaging, however, was limited because prolonged exposure alters device frequencies, causing a loss of injection‐locking and microscope synchronization. Importantly, these observations also provide an indirect probe of the radiation hardness of CoFeB/MgO‐based SHNOs, revealing how extended x‐ray exposure affects their magnetic properties. The possible origins and implications of this behavior are discussed below.

Naturally, the simplest explanation of the x‐ray‐induced alterations of the magnetic properties is radiation‐induced heating. To put this to the test, we reduced the photon flux on the sample. This allows any temperature gradient to dissipate before it can induce further thermal annealing in the CoFeB. However, the PMA changes solely depended on the dose (time‐integrated flux). Smaller doses and longer radiation intervals produce consistent degradation in all samples. Furthermore, the effect occurs sharply, while thermal annealing is a gradual process that requires a sample to be kept at high temperatures for a longer period of time. In addition, we confirmed that these devices can operate at room temperature for long periods of time without degrading, which excludes Joule heating as the origin of the effect. Thus, the observations of briefly modified properties followed by an irreversible degradation are only consistent with chemical changes induced by the x‐rays.

The AO frequency of SHNOs is closely related to the ferromagnetic resonance (FMR) [48], which is largely governed by the external field, saturation magnetization, and anisotropy [49, 50]. The anisotropy in our samples is dominated by the PMA induced at the CoFeB/MgO interface. This effect is achieved via symmetry breaking and hybridization of the Fe(Co)‐3d and O‐2p orbitals at the metal/oxide interface [51, 52, 53, 54]. In order to hybridize, the Fe and Co atoms should be in the crystalline Co‐Fe form, rather than the amorphous CoFeB. During the annealing process at fabrication, B diffuses into the HM metal layer, with Ta acting as a boron sink, which allows Co─Fe to form. The value of the PMA will depend on these two factors: the crystallinity at the metal/oxide interface and the B diffusion into the HM layer. By understanding the effect of the x‐rays on both, we can explain the irreversible changes induced during our measurements.

In our case, the electromagnetic radiation most likely modifies the metal/oxide interface. High doses of x‐rays can generate oxygen vacancies in the oxide at CoFeB/MgO [55]. The same ionizing process could also reduce Co and Fe oxides at the interface [56], allowing O to migrate back into the MgO, enhancing the crystallinity and thereby the PMA, leading to the effect shown in Figure 4 (higher $f_{\mathrm{AO}}$ and amplitude).

Another method to modify interfaces is ion bombardment. Devolder et al. studied the effect on Ta/CoFeB/MgO stacks, showing that small fluences mitigate B diffusion and enhance the PMA, likely due to CoFeB crystallization [57]. A similar process could be taking place in our samples, with the x‐rays generating both oxygen ions and photoelectrons that drive B diffusion. In larger doses, the radiation would cause a breakdown of the MgO due to oxygen vacancies, which is also consistent with our experiments.

Our observations challenge the often‐promoted claim of radiation hardness of CoFeB/MgO‐based spintronic devices (i.e., that x‐ray or ionizing radiation does not modify their magnetic properties) [58]. Radiation hardness is particularly relevant for technological applications of spintronic devices in high‐radiation environments, such as spacecraft, satellites, or particle accelerators, where exposure to ionizing radiation could unintentionally alter magnetic properties and compromise device performance. Additional studies are needed to fully clarify the mechanism of the x‐ray‐induced modification, which is of considerable interest given the scientific and technological significance of CoFeB/MgO heterostructures [59, 60]. SHNOs might be an ideal system for further studies, since the amplitude and frequency of their AOs are sensitive to small changes in the local effective magnetic field [17, 19, 29] and can be detected using electrical or time‐integrated optical techniques.

## 3 Conclusion

In summary, SHNOs are central in the advancement of scalable spintronic microwave devices. By using TR‐STXM, we have directly observed AO modes in an SHNO and recorded their time evolution with exceptional spatial resolution. The measurements were reproduced by micromagnetic simulations, which demonstrated that commonly overlooked parameters are, in fact, important. A fabrication‐induced reduction of PMA together with polycrystallinity (grains) displaces the AO modes and causes amplitude asymmetry between them, whereas DMI yields anisotropic SW propagation. In addition, we found that x‐ray radiation irreversibly and constantly modifies the effective anisotropy, making more in‐depth characterizations of CoFeB/MgO stacks challenging. Overall, the results show the need to revise the physical models underlying SHNOs and their mutual synchronization. These insights contribute to a better understanding of the individual and collective oscillation dynamics, with implications for accelerating the design of spintronic complex networks for applied next‐generation computing architectures.

## 4 Experimental and Computational Methods

### 4.1 Device Fabrication

${\mathrm{AlO}}_{x}$(3nm)/$W_{88}$ ${\mathrm{Ta}}_{12}$(5nm)/${\mathrm{Co}}_{20}$ ${\mathrm{Fe}}_{60}$ $B_{20}$(1.4nm)/MgO(2nm) stacks were grown on SiN‐coated p‐Si(100) substrates, containing membrane slits of size $20\times 300\;\;\mu m^{2}$ and thickness 200nm. The growth conditions for all layers were similar to those in previously reported works [22, 29, 61]. Subsequently, the samples were annealed at 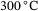 for 1 h to crystallize the CoFeB and MgO at the interface. Afterward, the stacks were patterned into SHNOs of three different nanoconstriction widths (120, 150, and 200 nm) using electron‐beam lithography (EBL) with the negative electron‐beam resist hydrogen silsesquioxane (HSQ, XR‐1541‐002, 2%). An Ar‐ion beam etching tool (Oxford Instruments Ionfab 300 Plus) was used to transfer the pattern. Next, the negative resist was removed, and ground‐signal‐ground (GSG) coplanar waveguide (CPW) masks were defined by an optical lift‐off lithography process. Finally, the CPW was deposited using a sputtered Cu(800nm)/Pt(20nm) bilayer. Due to the radiation‐induced irreversible changes mentioned above, only the 150nm device could be measured successfully using STXM.

### 4.2 Electrical Setup

For electrical characterization and injection‐locking of the device, the sample was connected to the following electrical setup. A sourcemeter (Keithley 2450 SMU) is used to supply a direct current $I_{\mathrm{DC}}$ to induce AOs in the nanoconstriction. The AO then induces an AC signal through the AMR effect. This signal passes the bias tee and a circulator, is amplified using a low‐noise amplifier (LNA) with 72dB gain, and finally detected using a spectrum analyzer (Agilent E4407B). To achieve injection locking, an arbitrary waveform generator (AWG) (Keysight M8195A) combined with a YIG bandpass filter and an amplifier was used to generate an AC signal with the desired locking frequency, which goes through the circulator and bias tee to reach the sample. IL is realized by twice the AO frequency (2f‐locking), which makes it possible to detect the increased AMR signal at 1f in the spectrum analyzer.

### 4.3 TR‐STXM Setup

The electrical setup was connected to the sample via a high‐frequency sample holder, which itself was positioned inside the vacuum chamber of the x‐ray microscope MAXYMUS at the BESSY II electron storage ring operated by the Helmholtz‐Zentrum Berlin für Materialien und Energie [62]. For TR‐STXM measurements, the sample was illuminated with monochromatic x‐rays from an undulator, with an energy corresponding to the Fe $L_{3}$ edge, making it possible to exploit the x‐ray magnetic circular dichroism (XMCD) effect to obtain magnetic contrast. Furthermore, the time structure of the synchrotron was utilized in an asynchronous pump‐probe scheme to achieve temporal resolution. This was used to directly observe magnetization dynamics on time and length scales required for nanoconstriction‐based SHNOs. A set of four rotatable permanent magnets allowed for controlling the magnetic field at the sample position, while a liquid helium‐flow cryostat enabled temperature control down to 20K.

### 4.4 Post‐Processing of TR‐STXM Measurements

The TR‐STXM measurements yield snapshots of the magnetization landscape at equidistant time steps, in this case 31 frames per dynamic cycle. To significantly increase the signal to noise ratio, these 31 frames were used to perform a fast Fourier transform (FFT), which transforms the signal into the frequency domain. The fundamental frequency was selected to eliminate noise at different frequencies, and a phase‐resolved snapshot was created by using the amplitude and phase of the Fourier transform. The dynamic representation of the data (animation in the Supporting Information) was retrieved by plotting the FFT data at different time steps. The evaluation of the data was similar to the procedure described in Ref. [63].

### 4.5 Micromagnetic Simulations

Micromagnetic simulations have been carried out using the GPU‐accelerated *mumax* solver [64], with the current density and Oersted field inputs calculated using the multiphysics COMSOL software [65], as shown in Section S.1. The SOT distribution is naturally set by the current density profile. The CoFeB layer was discretized using a 1024×1024×1 rectangular mesh with 4nm × 4nm × 1.4nm cell size and current and field geometry according to the experimental setup shown in Figure 1a. The SOT was modeled by a Slonczewski term using the input current density and a spin Hall angle of 0.6. The CoFeB layer parameters were chosen as: saturation magnetization $M_{S}=1050\;kAm^{-1}$, exchange stiffness $A_{\mathrm{ex}}=19\;pJm^{-1}$, Gilbert damping constant $\alpha_{0}=9\cdot 10^{-3}$, and gyromagnetic ratio $\gamma /2\pi =29.1\;G\mathrm{Hz}T^{-1}$, in line with previous work on similar bilayers [19]. The grain boundaries were simulated using different magnetization regions generated by Voronoi tessellation with an average diameter of 9nm, their PMA was modeled using a random normal distribution with an average of 0.92 of the measured PMA value and a width of 5%. The measured PMA was $645\;kJm^{-3}$. The interfacial DMI value was $250\;\mu J/\;m^{2}\;$, as reported in the literature [45]. To match the simulations with the measurements, we systematically explored several different combinations of grain size and PMA variation, and the ones we report here are the best fit for the observed phenomena. During this exploration, we did not come across any other combination of reasonable parameters to produce similar results. Therefore, we conclude that polycrystallinity and DMI play an important (and hitherto overlooked) role in AO dynamics in SHNOs. Note that the simulations describe free‐running AOs without injection locking. For comparison with the experiment, the simulated data were frequency‐filtered at 6.62 GHz, the frequency showing the strongest response.

## Conflicts of Interest

The authors declare no conflicts of interest.

## Supporting information


**Supporting File**: adma74547‐sup‐0001‐SuppMat.pdf.

## Data Availability

The data that support the findings of this study are available from the corresponding authors upon reasonable request.
