# Supplementary material for: Direct Observation of Propagating Spin Waves in a Spin Hall Nano‐Oscillator
Source: Adv Mater. 2026 Aug 28;38(52):e74547. doi: 10.1002/adma.74547 (PMC13579080; doi:10.1002/adma.74547)
Supplement: Supplementary file 1 [file ADMA-38-e74547-s001.pdf]

Supporting Information for *Direct Observation of Propagating Spin Waves in a Spin Hall Nano-Oscillator*

Victor H. González, Frank Schulz, Nilamani Behera, Martina Ahlberg, Akash Kumar, Andreas Frisk, Felix Groß, Sven Erik Ilse, Steffen Wittrock, Markus Weigand, Gisela Schütz, Johan Åkerman*, Sebastian Wintz**

S.1 Current and field distributions

The current and Oersted-field distributions play an important role in the dynamics of the SHNO. Therefore, we simulated them in high resolution using the COMSOL Multiphysics software and used the result as input for the micromagnetic simulations. A projection of the COMSOL results is shown in Fig. S1.

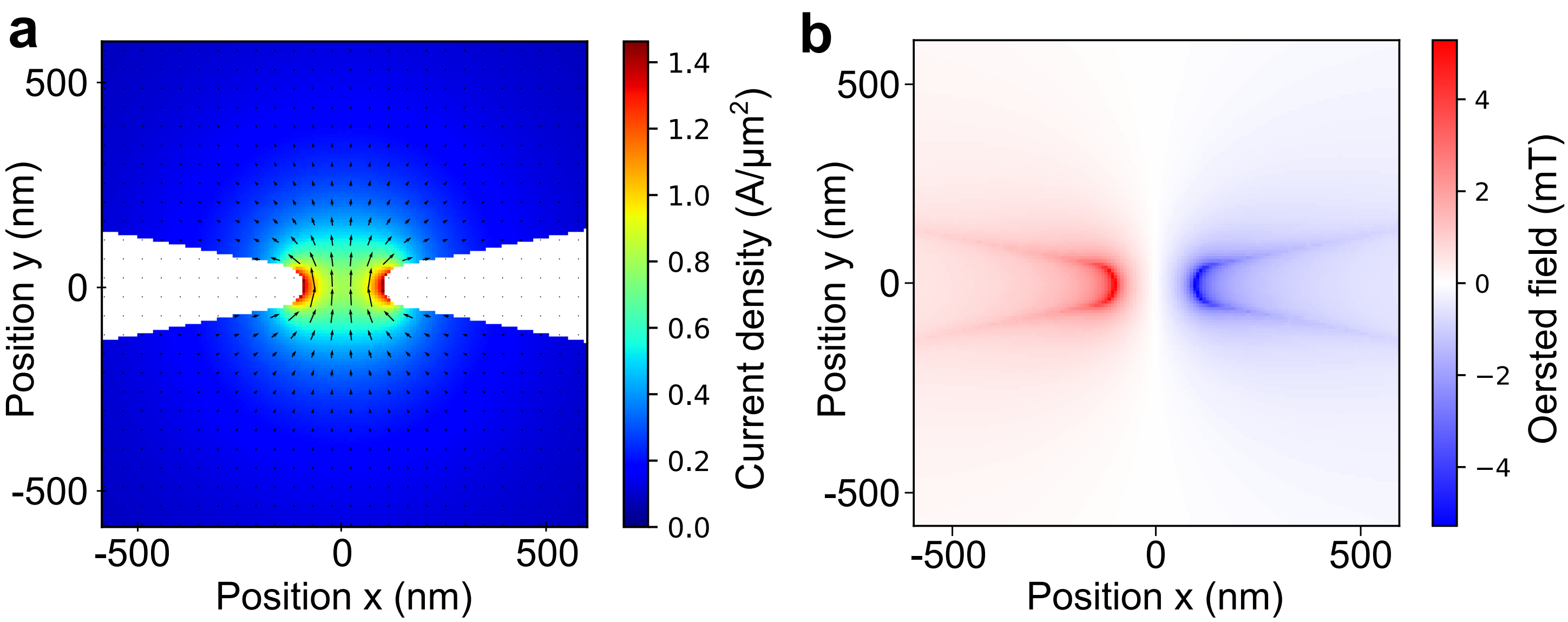

Figure S1: **Current and Oersted field distribution in the SHNO. a.** Current distribution. The direction and size of the arrows correspond to the current density vector at each point. **b.** Oersted field along the z direction, generated by the current flowing through the heavy metal and ferromagnetic layers across the constriction.

S.2 Animations of time-resolved magnetization dynamics

Movie #1 (experiment.gif) and Movie #2 (simulation.gif) show the time-dependent out-of-plane magnetization m_z at the nanoconstriction corresponding to the snapshots shown in Fig. 2 of the main text, as measured by TR-STXM and reproduced by micromagnetic simulations, respectively. The grayscale encodes the sign and relative magnitude of m_z, and the dark regions in the experimental movie mark the edges of the constriction. Both movies illustrate the strongly anisotropic spin-wave emission discussed in the main text. The experimental Movie #1 has a lateral size of 800 nm x 800 nm and the time step between consecutive frames is 6.74 ps. The simulated Movie #2 displays a larger area of 1350 nm x 1350 nm, the time step between frames in the animation is 10 ps, and it uses an iso-amplitude contrast representation.
